# Evolutionary analysis of *MOCA1* gene in *Arabidopsis thaliana* and functional comparison with *PGSIP7* and *PGSIP8* genes

**DOI:** 10.3389/fpls.2026.1734646

**Published:** 2026-02-24

**Authors:** Xinwen Hou, Shanmin Zhou, Yujun Guan, Huiyao Shi, Weizhong Liu

**Affiliations:** 1 School of Chemistry and Chemical Engineering, Shanxi Normal University, Taiyuan, China; 2 School of Life Science, Shanxi Normal University, Taiyuan, China

**Keywords:** *Arabidopsis thaliana*, *MOCA1*, *PGSIP7*, gene evolution, salt sensing

## Abstract

Soil salinization is becoming a more serious environmental issue. Excessive salt will hinder the growth and development of plants, reduce crop yields, limit global agricultural production, and increasingly threaten the sustainability of global food supplies. Salt sensing refers to the process in which external Na^+^ stimulates plants. GIPC binds Na^+^ to activate Ca^2+^ channels, leading to Ca^2+^ influx; this cytosolic Ca²^+^ signal is subsequently transmitted to the SOS signaling pathway, triggering intracellular Na^+^ efflux and vacuolar storage. The salt tolerance of plants is a trait that emerged gradually through evolutionary adaptation, rather than an inherent property of the first land plants. Although terrestrial plants appeared about 450–470 million years ago, the origin and evolution of plant salt sensing remain unclear. In this study, two potential salt-sensitive genes, *PGSIP7* and *PGSIP8*, in glycosyltransferase family 8 were identified through bioinformatics analysis. Further salt stress treatment found that the *PGSIP7* gene responded to salt stress, which showed limited growth and development and decreased Na^+^ transport capacity in the SOS pathway, while the *PGSIP8* gene was insensitive to salt stress. In addition, the origin and evolution of the *MOCA1* gene were preliminarily explored by cloning homologous genes of the *Arabidopsis* salt-sensing gene *MOCA1* from lower to higher plants to obtain transgenic plants. It was found that the *MOCA1* gene originated from the single-celled plant *Chlamydomonas reinhardtii*, and its homologous gene is *Cre10g422450v5*. The *MOCA1* gene has weak salt-sensing ability in lower plants, but with evolution, its ability to sense Na^+^ gradually increases in higher plants. Although the *MOCA1* gene has different salt sensitivity in different plants, its overexpression can improve salt tolerance. Our results have laid a certain experimental framework for subsequent investigations concerning the evolution of salt-sensing genes in plants and the identification of new salt-sensitive genes. They also provide research ideas for enhancing crops’ resistance against salt.

## 1 Introduction

Land salinization seriously restricts global food security and the ecological environment of countries around the world. Excessive salt restricts the growth and development of plants or even kills them, and endangers worldwide crop productivity (Chele et al., 2021; Hassani et al., 2021). Salinity stress is divided into continuous accumulation of Na^+^ stress and early osmotic stress. On the one hand, high salt concentrations increase the osmotic pressure of soil solutions, which hinders plant roots’ ability to absorb water, causing dehydration of plant cells and triggering osmotic stress. On the other hand, plants grown for a long time in soil with a high concentration of salt will accumulate Na^+^, resulting in ion imbalance and toxic effects and disrupting normal metabolic processes. These early osmotic and subsequent ionic insults are perceived primarily by the mechanosensitive Ca^2+^ channel *OSCA1* (Yuan et al., 2014) and the Na^+^-binding GIPC–*MOCA1* complex (Jiang et al., 2019), respectively, which together couple extracellular salt levels to downstream Ca^2+^ signaling and the SOS pathway. Salt stress destroys the chloroplast structure and affects the synthesis of photosynthetic pigments and photosynthetic electron transport, thereby reducing photosynthesis efficiency. Salt stress also impacts the functions of organelles, including endoplasmic reticulum and mitochondria, resulting in the obstruction of metabolic activities within cells. In addition, plant cells under salt stress can also induce more reactive oxygen species (ROS), and elevated ROS may oxidatively damage proteins, nucleic acids, and cell membranes. Long-term salt stress can have severe consequences on crop health, development, and yield.

Sodium input and sensing, the earliest response of cells to salt, is a complex process (Puri and Lee, 2021). The newly identified plasma-membrane Na^+^ sensor *MOCA1* (*monocation-induced [Ca^2+^]_i_ increases 1*) has been shown to mediate the perception of extracellular salt, including but not limited to Na^+^. Map-based cloning revealed that the protein IPUT1 (inositol phosphorylcreamide glucuronosyltransferase 1) encoded by the *MOCA1* gene is an enzyme that transfers GlcA (glucuronic acid) to the phosphate head group of IPC (inositol phosphorylcreamide, a ceramide-based plasma-membrane sphingolipid bearing a phosphoryl-inositol moiety) and facilitates the formation of GIPC (glycosyl inositol phosphorylceramide, its GlcA-capped variant presenting negatively charged carboxylates) on the outer leaflet. Under salt stress, the negatively charged sites on GIPC sphingolipids can bind to Na^+^, causing rapid changes in the cell surface potential, thereby opening the calcium channel connecting the inside and outside of the cell, making the intracellular calcium concentration increase rapidly. The increase of intracellular Ca^2+^ concentration further activates the SOS pathway. Activated SOS3 combines with SOS2 to form a protein complex, which activates protein kinase activity of the latter and then activates the plasma membrane’s Na^+^/H^+^ antiporter SOS1 in a phosphorylated manner, transporting excess Na^+^ outside the cell, activating related salt metabolic reactions, further regulating cell physiological and biochemical activities, and helping plants adapt to salt stress environments (Chen et al., 2023).

GIPC is the main sphingolipid in the plasma membrane of plants and fungi and is also one of the most abundant sphingolipids in terrestrial organisms. Plant GIPC contains a polar head structure and a ceramide moiety, and its core structure usually includes a trihydroxylated LCB part connected to inositol-glucuronic acid via a 2-hydroxylated very long-chain fatty acid. Through a series of specific enzymatic reactions, glycolipids similar to GIPC Series A (such as GlcR1-GlcA-inositol-1-phosphoceramide) or GIPC Series B (such as Gal-GlcR1-GlcA-inositol-1-phosphoceramide) can be formed. In general, polysaccharide structures associated with inositol-phosphoceramide (IPC) fall into two categories: hexose-conjugated IPC (e.g., GlcA-IPC) in the vegetative tissues of *Arabidopsis*, and N-acetylglucosamine-conjugated IPC (e.g., GlcNAc-IPC) in the reproductive tissues (seeds and pollen) of *Oryza sativa*, *Nicotiana tabacum*, and *Arabidopsis*. In *Nicotiana tabacum*, *IPUT1* overexpression or silencing led to changes in IPC glucuronosyltransferase activity, respectively, which is a direct precursor for GIPC production. Plants that silence *IPUT1* accumulated IPC as well as ceramide and glucosylceramide, while plants that overexpress *IPUT1* showed higher GIPC content. The fact that pollen cannot transmit *IPUT1* mutations suggests that these sphingolipids are vital to plants (Rennie et al., 2014).

The *MOCA1* gene, also known as *Plant Glycogenin-like Starch Initiation Protein 6* (*PGSIP6*), belongs to the glycosyltransferase family. It is a large superfamily of plant genes. In *Arabidopsis*, there are 361 members divided into 28 subfamilies. The family members are very diverse. In plants, the *GT8* subfamily includes the *Glycosyltransferase Family 8 Xylan Glucuronosyltransferase* (*GUX*), *Galactitol Synthase* (*GolS*), *Galacturonosyltransferase* (*GAUT*), and *Galacturonosyltransferase-like* (*GATL*) branches, among others (Yin et al., 2010; Zhang et al., 2024). These branches do not include the three *Arabidopsis* GT8 proteins: PGSIP6, PGSIP7, and PGSIP8. The *PGSIP* gene is an alpha-glucosyltransferase involved in initiating starch synthesis, which requires glycosylation of proteins and oligosaccharides. It was discovered that *AtPGSIP1* and *AtPGSIP3* participate in secondary cell wall synthesis (Yin et al., 2010). The synthesis of disaccharides, oligosaccharides, and polysaccharides is inseparable from the catalysis of up to a hundred different glycosyltransferases (GTs) in cells. These transferases with different functions catalyze the activation of the donor and transport it to specific acceptor molecules to generate glycosidic bonds. Unlike classical GT8 enzymes that utilize UDP-sugars to build or decorate cell-wall polysaccharides, PGSIP proteins are autoglucosyltransferases that function specifically in starch initiation.

In order to adapt to the terrestrial environment, large-scale evolution occurred in the process of plant diversification from simple green algae to complex multicellular terrestrial plants (Zhang et al., 2020b). Lower plants, especially mosses and ferns, control Na^+^ contents in the body by controlling inflow and outflow. However, Na^+^-ATPase does not seem to be expressed in higher plants (Chen et al., 2022), and they transport excess Na^+^ out of the cytoplasm or store it in vacuoles through Na^+^/H^+^ antiporters (Xie et al., 2022; Wang et al., 2023). Angiosperms show an extensive scope of salt tolerance, ranging from low salt concentrations that inhibit growth (for example, 25 mM NaCl kills the sensitive chickpea genotype) (Flowers et al., 2010) to tolerance concentrations equal to or above seawater (80 mM NaCl, soil solution conductivity of 7.8 dsm^-1^), which are classified as either halophytes or non-halophytes (salt-sensitive plants) (Yuan et al., 2019), and about 1550 salt-tolerant plants are listed. The order with the largest number of halophytes is Caryophyllales (including some plants belonging to the Chenopodiaceae) (Matinzadeh et al., 2019). These halophytes have evolved specific salt glands to remove excess salt to adapt to high-salt environments. Plant cell walls regulate cell shape under high-salinity conditions, thereby maintaining normal physiological functions according to their own tension generated by the osmotic pressure produced. Therefore, the formation of cell walls is particularly important. Plant glycosyltransferases are one of the key enzymes in cell wall synthesis. Rhodophyta and Chlorophyta usually have fewer glycosyltransferases than terrestrial plants. The *GT8* glycosyltransferase family has a long history that evolved prior to the differentiation of all bacterial phyla, possibly billions of years ago (Yin et al., 2010). The *PGSIP* branch in the *GT8* subfamily may have been acquired from archaea to plants through horizontal gene transfer (Yin et al., 2010).

However, the specific roles of *PGSIP* branch genes in plant salt tolerance remain largely unexplored. Given their involvement in cell wall synthesis and the critical role of cell walls in salt stress adaptation, we hypothesize that *PGSIP* branch genes contribute to plant salt tolerance. To test this hypothesis, this study aims to (i) characterize the function of *MOCA1*, *PGSIP7*, and *PGSIP8* in salt tolerance by analyzing phenotypes, ROS content, the expression of salt-responsive genes, and calcium ion signaling in corresponding mutant and transgenic plants under varying salt stress levels, and (ii) explore the origin and evolution of the *MOCA1* gene and its acquisition of salt-sensing function. These efforts are expected to identify potential salt-sensitive genes and provide a foundation for investigating the evolution of salt sensing from lower to higher plants and for improving salt tolerance in crops.

## 2 Materials and methods

### 2.1 Plant materials and growth conditions

*Arabidopsis* (Columbia-0) and the Columbia-0 seed line that constitutively expresses the intracellular Ca^2+^ indicator aequorin are preserved in our laboratory. Mutants of *pgsip7* (Salk_016683C), *pgsip8* (Salk_037413C), and *sos2* (Salk_063265C) were purchased from AraShare. The *moca1* mutant and *p35S::AtMOCA1* were provided by Professor Zhenming Pei. *Chlamydomonas reinhardtii*, *Physcomitrella patens*, and *Oryza sativa* were preserved in our laboratory, and transgenic plants were constructed for this experiment. A growth chamber with controlled conditions (25 °C day/22 °C night, 16 h light/8 h dark, 110 µmol m^-2^ s^-1^ white light) was used for cultivating plants.

### 2.2 Green cotyledon ratio and root length determination

The green cotyledon ratio (RGC) can reflect the degree of stress.  Green Cotyledon Ratio=Green Cotyledon Plants/Total Plants×100%. *Arabidopsis* seeds were planted on 1/2 MS medium with varying doses of sorbitol (0–400 mM) and NaCl (0–150 mM) and photographed ten days later. The concentration of 60 mM NaCl was selected as the threshold based on preliminary gradient experiments, as this was the minimal concentration that induced significant phenotypic differences between wild-type and salt-sensitive mutants without causing non-specific inhibition. *Arabidopsis thaliana*’s root length was determined using ImageJ (Zhou et al., 2011).

### 2.3 Searching for protein sequences of *MOCA1* homologous genes

The *Arabidopsis* MOCA1 protein sequence was used as a reference, and the conserved domains in the MOCA1 protein sequence were used as the criterion. Download the whole proteome sequences of *Chlamydomonas reinhardtii* (v5.6), *Physcomitrella patens* (v3.3), and *Oryza sativa* (v7.0) from the Phytozome v13 database (https://phytozome-next.jgi.doe.gov/), and use BioEdit to establish a local protein library and BLAST-P to search for potential homologous genes. The TAIR database (https://www.arabidopsis.org/) provided the MOCA1 protein sequence of *Arabidopsis*.

### 2.4 Bioinformatics analysis

Information on members of the *Arabidopsis GT8* subfamily is provided by the TAIR website. The MAFFT (https://mafft.cbrc.jp/alignment/server/) was applied to align protein sequences using the FFT-NS-I algorithm (Katoh and Standley, 2013). The amino acid sequence scoring matrix parameter was BLOSUM62. The neighbor-joining (NJ) method was then used to perform phylogenetic analysis on the aligned sequences using MEGA X with 1000 bootstrap replicates (Kumar et al., 2018). Transcriptome data of *GT8* subfamily members were obtained from the ePlant (http://bar.utoronto.ca/eplant_soybean/). The FPKM values were normalized, and HemI was utilized to create a heat map of expression (Deng et al., 2014). The alpha helices of the MOCA1, PGSIP7, and PGSIP8 protein structures were predicted by the Araemnon (http://aramemnon.botanik.uni-koeln.de). The protein tertiary structure was predicted by the SIWSS-Model and optimized by ChimeraX 1.3. Ensembl (https://plants.ensembl.org/index.html) is the source of information about *MOCA1* homologous genes. The sequences were aligned using MAFFT, and further visualized using Jalview software.

### 2.5 Quantitative real-time PCR

Total RNA was obtained with TransZol (TransGen Biotech, Beijing, China), and cDNA was prepared by EasyScript^®^ First-Strand cDNA Synthesis SuperMix (TransGen Biotech, Beijing, China). The primers used were designed using Primer 5.0 according to standard primer design principles (**Supplementary Table S1**). Using the 2× Perfect SYBR Green PCR Mix (TransGen Biotech, Beijing, China) and the QuantStudio 3 PCR System (Life Technologies, Singapore), qRT-PCR was carried out rigorously, adhering to the manufacturer’s instructions. Relative gene transcript levels were measured as 2^−⊿⊿Ct^, and ACTIN served as an internal control (Li et al., 2019).

### 2.6 ROS accumulation in the main roots of *Arabidopsis*

ROS produced by xanthine dehydrogenase 1 (AtXDH1) in *Arabidopsis* were detected by nitroblue tetrazolium (NBT) and 3,3’-diaminobenzidine (DAB) staining. The *Arabidopsis* roots were immersed in NBT solution (0.1%, pH 7.8) and vacuum infiltrated for 20 minutes to allow the solution to fully penetrate the root tissue, incubated in the dark for 15 minutes, and washed with 95% ethanol to remove chlorophyll. The production of superoxide anions was assessed by observing the distribution of blue precipitates under a microscope. The roots of *Arabidopsis* were immersed in DAB solution (1 mg/mL, pH 3.8) and vacuum infiltrated for 20 minutes, incubated in the dark for 45 minutes, and decolorized with 95% ethanol. Hydrogen peroxide production was assessed by observing the distribution of brown precipitates under a microscope. For quantitative analysis, grayscale analysis was performed using ImageJ software (National Institutes of Health, USA). The mean gray value of the stained area was measured to quantify ROS accumulation levels. Briefly, the color images were converted to 8-bit grayscale, and the mean gray value was calculated after subtracting the background. Each experiment was repeated three times, with no less than 10 roots selected per group.

### 2.7 Aequorin bioluminescence-based Ca^2+^ imaging

*Arabidopsis* that expresses aequorin was used to assess the cellular free Ca^2+^ concentration ([Ca^2+^]_i_) (Knight et al., 1991; Yuan et al., 2014). In a 150 mm × 150 mm petri dish, 3.3 mL of 10 mM coelenterazine was equally sprayed on nine-day-old seedlings and left in the dark for 12 hours. A ChemiPro HT system equipped with a cryocooled, back-illuminated CCD (charge-coupled device) camera and a light-tight box was used for imaging. WinView/32 (Roper) controls the camera. Treat seedlings with either water or 200 mM NaCl, and take photos every four minutes to record fluorescence values. The seedlings were then exposed to strong light for one minute and photographed for two minutes to record chlorophyll spontaneous fluorescence. The experiments were conducted in the dark. Luminous images were analyzed using MetaMorph.

### 2.8 Plasmid construction and plant transformation

The target fragments were amplified from cDNA libraries of different plant materials. The homologous genes of the *MOCA1* gene in *Chlamydomonas reinhardtii*, *Physcomitrella patens*, and *Oryza sativa* are *Cre_10g422450v5*, *Pp3c2_32110*, *Pp3c1_6500*, and *loc_Os02g41520.1*, respectively. A complete circular plasmid was constructed by ligating the target sequence to the linear vector, leveraging the complementarity between the restriction enzymes at both ends of the sequence and those at both ends of the linear vector. After transforming the recombinant plasmid into *Agrobacterium* GV3101, we applied the floral dip method to transform the *Arabidopsis* mutant *moca1*. T0 seeds were planted on 1/2 MS medium (HygB^+^) and transplanted after growing four true leaves. Plants that grew normally on the antibiotic-containing selective medium were T1 generation positive transgenic plants.

### 2.9 GUS tissue staining

The principle of GUS staining is that under appropriate reaction conditions, β-glucuronidase (GUS) could react with 5-bromo-4-chloro-3-indolyl-β-D-glucuronide (X-Gluc) to produce insoluble blue substances, 5,5’-dibromo-4,4’-dichloroindigo, which are visible under a microscope. The 1500 bp sequence before the ATG of the *CreMOCA1* gene’s start codon was selected to construct the *proCreMOCA1::GUS* vector and further obtain transgenic *Arabidopsis* plants. Seven-day-old seedlings of *proCreMOCA1::GUS* transgenic *Arabidopsis* were exposed to NaCl (0, 50, and 100 mM) for 6 hours and then completely immersed in the GUS staining solution. After incubation at 37 °C in the dark for 2 hours and decoloring with 70% ethanol, the activity sites of GUS showed blue.

### 2.10 Statistical analyses

Statistics were analyzed using SPSS. Statistical significance was evaluated using two-way ANOVA. *P < 0.05, **P < 0.01, and ***P < 0.001. Results were expressed as mean ± s.d. Each experiment was conducted three times independently.

## 3 Results

### 3.1 Classification and tissue expression of the *GT8* subfamily

**Figure 1:**
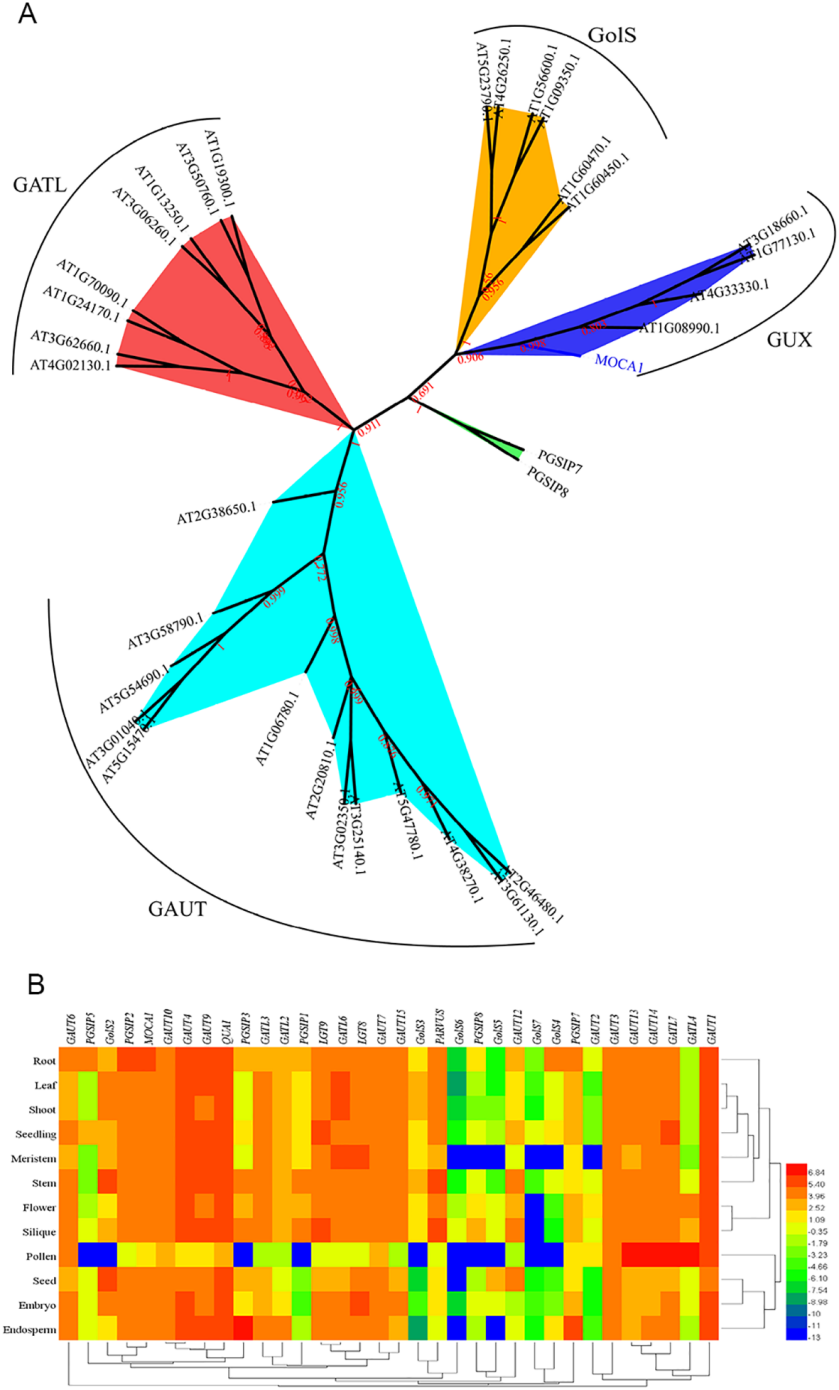
Classification and tissue expression of *GT8* subfamily. **(A)** The evolutionary relationships between the *GT8* subfamilies. The evolutionary tree was constructed based on amino acid sequences, and sequence alignment was performed using the MAFFT online platform. After alignment, MEGA X was used to build the evolutionary tree by the neighbor-joining (NJ) method with 1000 bootstrap replicates. Different colors are used to represent different classifications, and each subfamily’s name is also indicated in the corresponding place. **(B)** Heat map of tissue expression levels in *GT8* subfamily genes. Red indicates high levels; blue indicates low levels.

The *MOCA1* gene belongs to the *glucuronosyltransferase subfamily 8* (*GT8*), which contains 34 members, divided into the *GUX*, *GolS*, *GAUT*, and *GATL* branches (Yin et al., 2010). Among them, *MOCA1* is in the *GUX* branch, while *PGSIP7* and *PGSIP8* are not in these four branches, which are separate branches (**Figure 1A**). The *PGSIP* gene is an alpha-glucosyltransferase involved in initiating starch synthesis. Before being discovered as a salt sensor, the *MOCA1* gene was annotated as *PGSIP6* and serves a major part in low manganese resistance in *Arabidopsis* (Bian et al., 2018), which means that it may have multiple functions as a sensor. *PGSIP7* and *PGSIP8* are evolutionarily closer to *MOCA1* than other members, so they may have similar functions. The transcriptome databases’ (Zhang et al., 2020a) cluster analysis showed that the expression levels of this gene family’s members differ across tissues (**Figure 1B**). The *GAUT1* gene has the highest expression in all tissues, while the *GolS7* gene has the lowest expression. The *MOCA1* gene was highest expressed in the roots, the *PGSIP7* gene was highest in the roots and endosperm, and the *PGSIP8* gene was highest expressed in the siliques. Each gene showed differential expression in *Arabidopsis* tissues, indicating that genes in this family have different functions and may be redundant.

### 3.2 Expression and protein tertiary structure of *PGSIP7*, *PGSIP8*, and *MOCA1* genes

**Figure 2:**
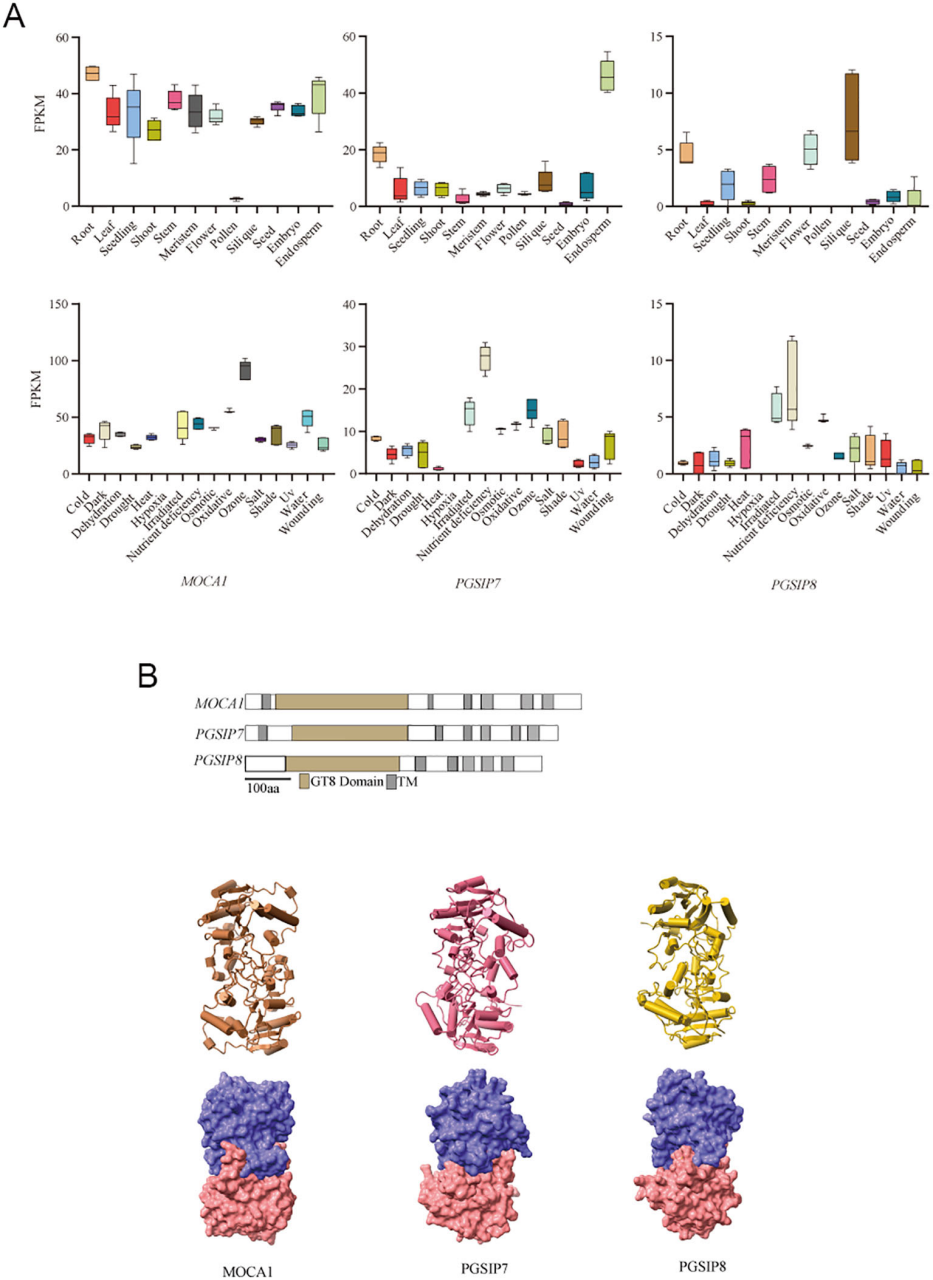
Gene expression and protein structure of *MOCA1*, *PGSIP7*, and *PGSIP8*. **(A)** Tissue and abiotic stress expression of *MOCA1*, *PGSIP7*, and *PGSIP8* genes. The above shows the expression levels of *MOCA1*, *PGSIP7*, and *PGSIP8* genes in various tissues of *Arabidopsis*. Below is the expression of *MOCA1*, *PGSIP7*, and *PGSIP8* genes under abiotic stress. **(B)** Secondary and tertiary structures of MOCA1, PGSIP7, and PGSIP8 proteins. The above are the secondary structures of three protein sequences, with the gray area representing the alpha helix region and the brown area representing the GT8 family conserved domains. Below is the tertiary structure of the protein, which consists of alpha helix model and protein surface model.

Transcriptome sequencing data revealed that *PGSIP7*, *PGSIP8*, and *MOCA1* gene expression levels in various tissues of *Arabidopsis* are shown in **Figure 2A**. Compared with the other two genes, the *MOCA1* gene has higher expression in various tissues, especially in seeds, embryos, and endosperm, suggesting that the *MOCA1* gene is crucial for germination. In roots, all three genes were highly expressed. Further investigation of these three genes’ expression under different abiotic stresses, including salt, cold, and drought, revealed that the *MOCA1* gene is sensitive to common abiotic stresses, the *PGSIP7* gene has high expression of nutrient deficiency, irradiation, and ozone, and the *PGSIP8* gene has high expression of nutrient deficiency, irradiation, and oxidation. The three genes’ expression varies in tissues and abiotic stress. *MOCA1* is the first salt-sensing gene discovered, and the *PGSIP7* and *PGSIP8* genes also have certain expressions under salt stress. **Figure 2B** illustrates that the MOCA1 protein sequence is the longest, while the PGSIP8 sequence is the shortest. Both MOCA1 and PGSIP7 proteins contain six helices, while PGSIP8 only contains five helices. Tertiary structure prediction of the protein sequences showed that their tertiary structures were highly similar, both consisting of two chains, A and B. The two subunits were tightly combined and contained multiple alpha helices, indicating that they may have similar functions.

### 3.3 Responses of *pgsip7*, *pgsip8*, and *moca1* to salt stress

*Arabidopsis* T-DNA insertion mutants were identified by the tri-primer method, and the primer information is shown in **Supplementary Table 2**. The amplification product in *Col-0* had only long fragments and no short fragments, while the three mutants had only short fragments and no long fragments, proving that all three mutants were homozygous mutants (**Supplementary Figure 1A**). According to information analysis in the TAIR database, it was found that the T-DNA insertion site of the *pgsip7* mutant was located in the third intron region of the gene, while the T-DNA insertion site of the *pgsip8* mutant was located in the first intron region of the gene, and the *moca1* mutant had a loss of a 12 bp fragment in the ninth exon region. Further experiments at the transcriptional level showed a significant decrease in gene expression in the mutants compared to the wild type (**Supplementary Figure 1B**).

**Figure 3:**
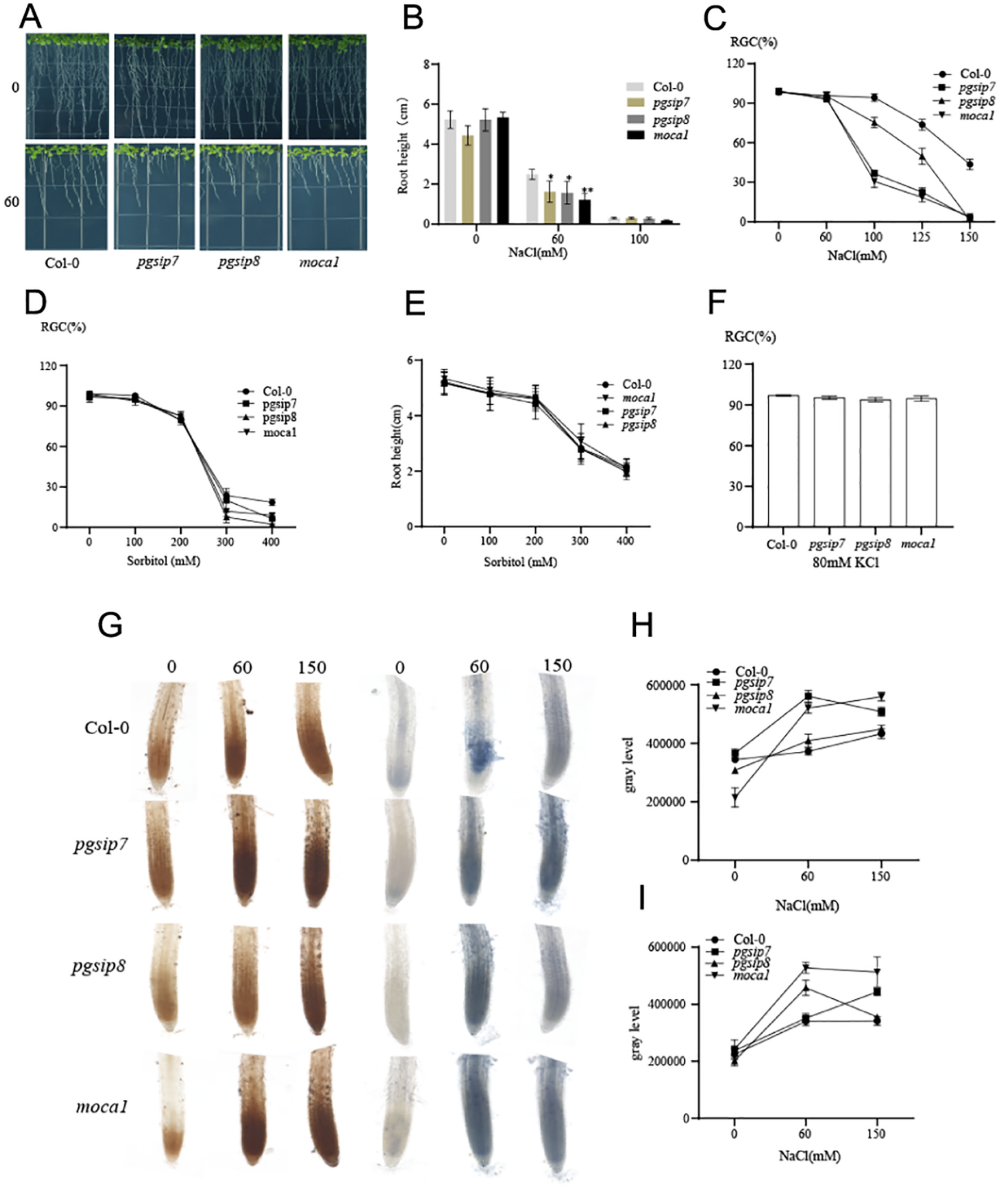
Different sensitivities of *moca1*, *pgsip7*, and *pgsip8* to salt stress. **(A)** The growth phenotypes of *moca1*, *pgsip7*, and *pgsip8* mutants were examined under 0 and 60 mM NaCl treatments. **(B, C)** Root length and cotyledon development of different genotypes of *Arabidopsis* under salt stress. The values in **(B)** are the mean ± s.d. of three independent replicates. (*P* < 0.05). RGC, the ratio of green cotyledons. **(D, E)** Effect of different concentrations of sorbitol on cotyledon development and root length of *Arabidopsis* with different genotypes. The values in **(C, D)** are the mean ± s.d. of three independent replicates (*n* = 90). **(F)** The effect of 80 mM KCl on cotyledon development of *Arabidopsis*. **(G)** ROS accumulation in the main roots of *Arabidopsis* under different salt stress concentrations for *moca1*, *pgsip7*, and *pgsip8* mutants. DAB staining on the left and NBT staining on the right. **(H)** The grayscale value of DAB staining. **(I)** The grayscale value of NBT staining. Grayscale analysis is performed using ImageJ, with each experiment repeated three times and no less than 10 roots selected.

Under varying concentrations of salt stress, the three mutants exhibited different extents of growth inhibition. The roots of plants were first inhibited by excessive Na^+^ under salt stress. When measuring root length, it was found that the root length of *moca1*, *pgsip7*, and *pgsip8* mutants was significantly inhibited under 60 mM salt concentration (**Figures 3A, B**). RGC index testing found that *moca1* and *pgsip7* were more salt-sensitive than the *pgsip8* mutant (**Figure 3C**). All three mutants were insensitive to osmotic stress and potassium stress (**Figures 3D-F**). When subjected to salt stress, plants will quickly respond to changes in their surroundings and regulate gene expression through intracellular signal transduction, among which ROS signal plays a very important role (Hasanuzzaman and Fujita, 2022; Kesawat et al., 2023; Zhou et al., 2024). The accumulation of ROS in the roots of the three mutants varied significantly under different salt stress concentrations (**Figures 3G-I**). Superoxide anions and hydrogen peroxide are accumulated in the roots of the *moca1* mutant, which increases with increasing salt concentration. It may be that the *moca1* mutant is insensitive to Na^+^, the normal ROS signal transduction pathway is affected, and the expression of related genes is suppressed, causing the ROS produced by stress not to be promptly cleared. Superoxide anions in the roots of the *pgsip7* mutant also accumulated with the increase of salt concentration. In contrast, superoxide anions in the roots of wild-type and *pgsip8* mutants did not accumulate with increasing salt concentration but were constantly cleared to maintain normal growth. ROS plays an important regulatory role as a signal molecule. Insensitivity to ROS signaling in *moca1* and *pgsip7* mutants will inhibit growth and development.

### 3.4 Salt-responsive gene expression in *pgsip7*, *pgsip8*, and *moca1*

**Figure 4:**
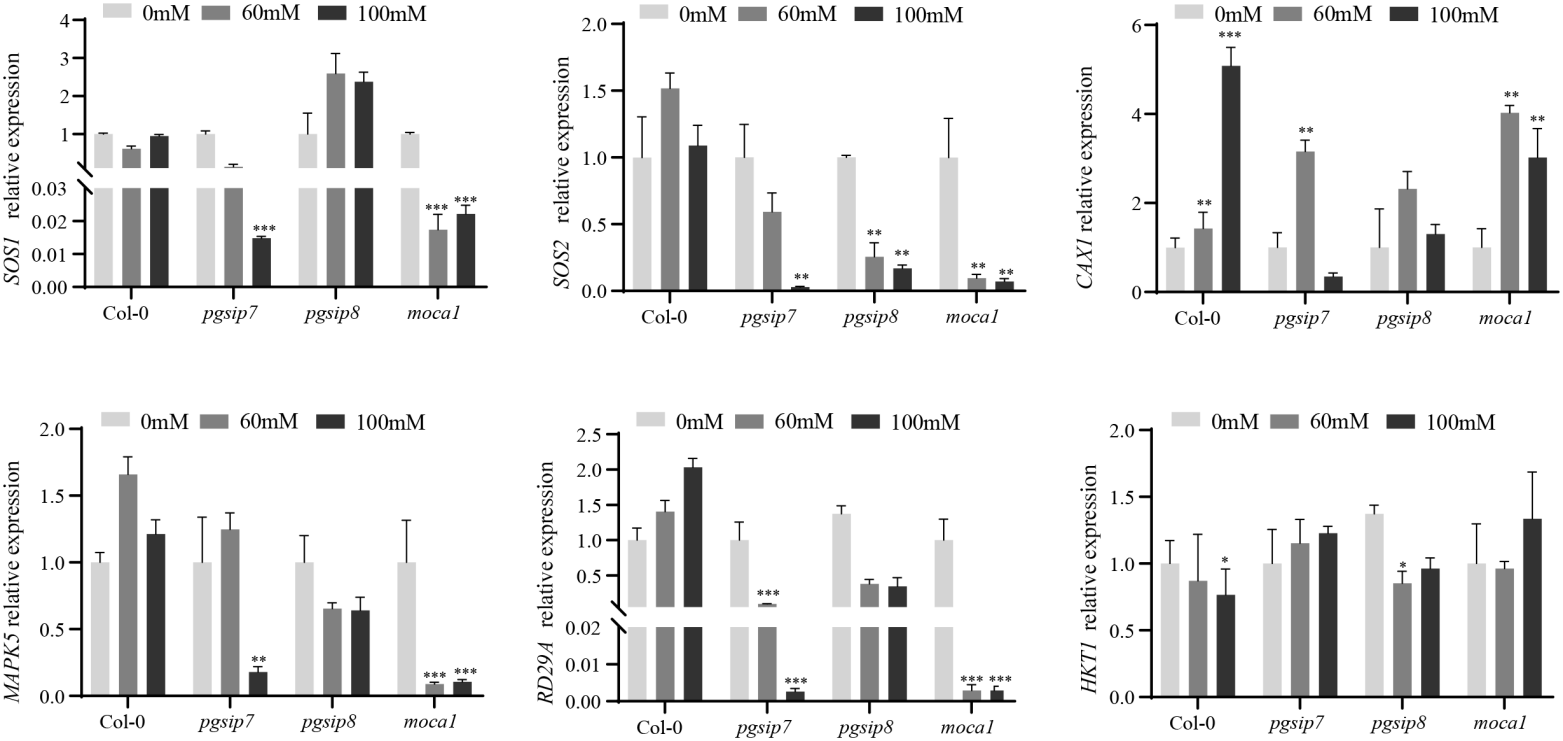
Salt-responsive gene expression in *pgsip7*, *pgsip8*, and *moca1*. The relative expression levels of six salt-responsive genes in *pgsip7*, *pgsip8*, and *moca1* mutants under salt stress were detected using qRT-PCR. Seven-day-old *Arabidopsis* plants were treated with different concentrations of NaCl (0, 60, and 100 mM) for 6 hours to analyze the expression patterns of salt-responsive genes. The expression level of *Col-0* was set to 1 at 0 mM NaCl. Each group of experiments was repeated three times. The data represent the mean ± s.d. *P < 0.05, **P < 0.01, ***P < 0.001.

Upon salt stress, multiple genes, including *HKT1*, are involved in the stress response to alleviate adverse impacts (Gholizadeh et al., 2024; Liu et al., 2024). Among them, the SOS signal transduction pathway, MAPK signal transduction pathway, and ABA signal transduction pathway have been studied in depth. We selected some key genes in these pathways for testing (**Figure 4**) (Ndathe et al., 2022; Zhang et al., 2023b; Liu et al., 2024). *SOS1* encodes a plasma membrane Na^+^/H^+^ antiporter. In wild-type plants, *SOS1* expression remained relatively stable across salt concentrations. In the *moca1* mutant, *SOS1* expression was significantly suppressed, consistent with its salt-hypersensitive phenotype. The *pgsip7* mutant showed decreased *SOS1* expression under salt stress, whereas the *pgsip8* mutant exhibited elevated *SOS1* expression with increasing salt concentration. *SOS2*, a protein kinase that interacts with SOS3 to activate SOS1, showed moderate induction in wild-type plants under salt stress. However, *SOS2* expression was severely suppressed in both *pgsip7* and *moca1* mutants, particularly at 100 mM NaCl. *CAX1* (Cation Exchanger 1), a vacuolar Ca^2+^/H^+^ antiporter, was significantly upregulated in wild-type plants under salt stress, peaking at 100 mM NaCl. By contrast, *CAX1* expression in *moca1* and *pgsip7* mutants displayed a transient upregulation followed by downregulation, indicating disrupted calcium signaling in these mutants. *MAPK5* (Mitogen-Activated Protein Kinase 5), a key component of the MAPK cascade, showed a moderate up-regulation in wild-type plants at 60 mM NaCl. Both *moca1* and *pgsip7* mutants exhibited severe suppression of *MAPK5* expression under salt stress, particularly at 100 mM NaCl. The *pgsip8* mutant also showed reduced *MAPK5* expression relative to wild type, but the suppression was less severe than in *moca1* and *pgsip7*. *RD29A* is sensitive to salt (NaCl and KCl) and non-ionic hyperosmotic stresses (Justamante et al., 2025; Zhou et al., 2025). Compared with wild type, the expression of the *RD29A* gene in *moca1* and *pgsip7* mutants exhibited severe suppression with increasing salt concentration, implying that they might be incapable of responding to salt stress signals, resulting in reduced tolerance in high-salt environments. *HKT1* (High-Affinity K^+^ Transporter 1), responsible for xylem Na^+^ unloading and Na^+^/K^+^ homeostasis, showed relatively minor expression variations across genotypes. Wild-type and *pgsip8* plants exhibited slight *HKT1* downregulation under salt stress, while *pgsip7* and *moca1* mutants showed slight upregulation at 100 mM NaCl. *Moca1* and *pgsip7* mutants displayed highly correlated expression patterns, whereas *pgsip8* showed distinct responses. This suggests that *PGSIP7* and *MOCA1* may function in a common regulatory module, distinct from *PGSIP8*. Collectively, these findings indicate that *PGSIP7* positively regulates salt stress responses, potentially through modulation of the SOS signaling pathway.

### 3.5 Response of *pgsip7 sos2 and pgsip8 sos2* double mutants to salt stress

**Figure 5:**
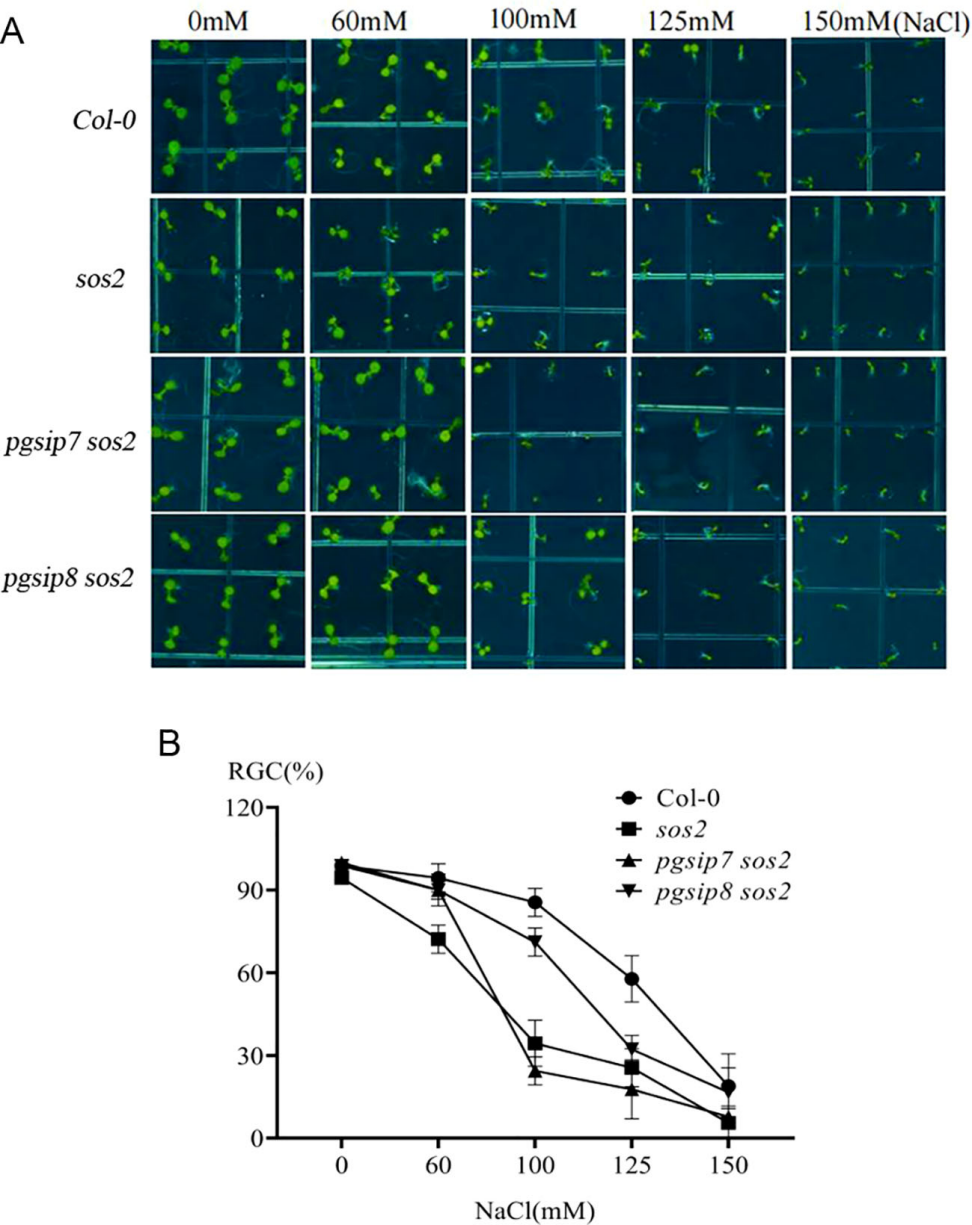
Role of the *PGSIP7* and *PGSIP8* genes in the SOS signaling pathway. **(A)** The *Col-0*, *sos2*, *pgsip7 sos2*, and *pgsip8 sos2* lines were treated with different salt concentration gradients (0, 60, 100, 125, and 150 mM) to investigate the development of cotyledons under salt stress. **(B)** Quantitative analysis of cotyledon greening of plants grown on 1/2 MS medium containing different concentrations of NaCl. RGC, ratio of green cotyledons. The values are mean ± s.d. of three independent repeats (*n* = 90).

In order to further confirm that the *PGSIP7* gene may be involved in the transport of Na^+^, F2 generation hybrid double mutant plants *pgsip7 sos2* and *pgsip8 sos2* were treated with different salt concentration gradients (**Figure 5**). Quantification of cotyledon greening showed that at 60 mM NaCl all lines remained ≥ 70% green, whereas at 100 mM the ratio for *pgsip7 sos2* fell to 24%—significantly below Col-0 (86%) and *pgsip8 sos2* (71%)—indicating lower salt tolerance. No difference was detected between the *pgsip8 sos2* double mutant and the wild type. The *sos2* single mutant attained only 34% green cotyledons and failed to expand; *pgsip8 sos2* partially rescued this phenotype, suggesting that the *PGSIP8* gene is not required for the SOS response, whereas *PGSIP7* may participate in Na^+^ transport within the SOS signaling pathway.

### 3.6 Evolution of the *MOCA1* gene

**Figure 6:**
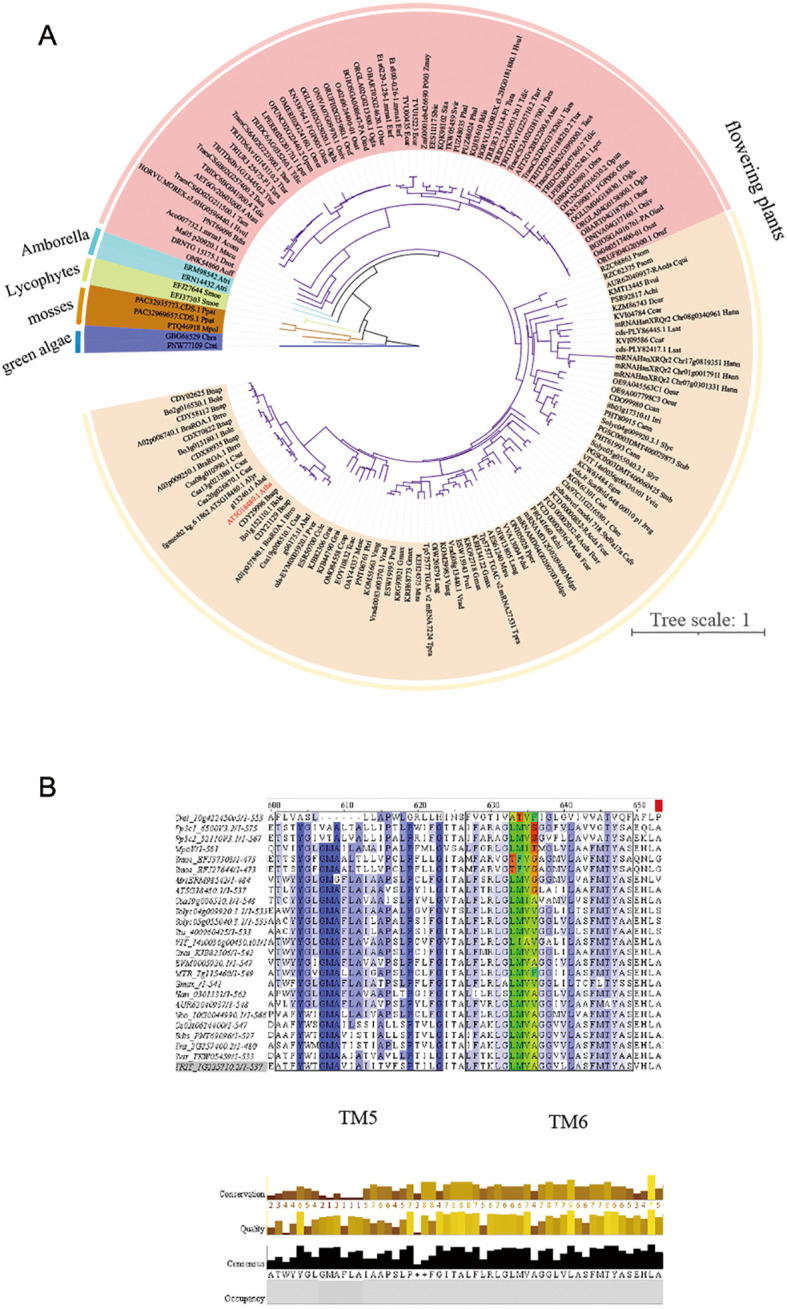
Evolution of the *MOCA1* gene. **(A)** Phylogenetic tree of the *MOCA1* gene in green algae, mosses, lycophytes, Amborella, and flowering plants. The homologous protein sequences of *Arabidopsis* MOCA1 protein in different species were obtained from the Ensembl database. The MAFFT online program was used to align all of the protein sequences in this study. MEGA X with the neighbor-joining method was then used to perform phylogenetic analysis. **(B)** Protein sequence alignment of *MOCA1* homologous genes in multiple species. TM5 and TM6 are two consecutive α-helix regions. The green region represents four conserved amino acid key regions that are aligned with the *Arabidopsis* MOCA1 protein across multiple species.

The *MOCA1* gene is the first salt-sensing gene found in *Arabidopsis thaliana*, and its gene function may have been obtained through gradual evolution. The evolutionary analysis from lower to higher plants is shown in **Figure 6A**. The homologous gene of the *MOCA1* gene first appeared in *Chlamydomonas reinhardtii*, a single-celled aquatic plant. As terrestrial plants emerged, the *MOCA1* gene appeared in *Physcomitrella patens*. We speculate that the *MOCA1* gene may have early incomplete salt-sensing ability at this time. As organisms on earth have experienced species explosion, plants continue to evolve and develop. The homologous genes of the *MOCA1* gene generally appear in higher plants, including *Oryza sativa*, potato, *Nicotiana tabacum*, and *Arabidopsis*, among others, giving them salt-sensing ability. The *MOCA1* gene evolves continuously as plants adapt to surroundings, from a single species at lower levels to multiple species at higher levels, showing rapid expansion.

During evolution, species may gradually change from one gene to multiple genes to adjust to their surroundings (Ebadi et al., 2023). The internal core segment of a gene generally does not mutate during evolution, but one or more amino acid sites will change as the environment changes (Dunham and Beltrao, 2021). The *MOCA1* gene is a key gene for salt sensing, and the evolution of key sites is particularly important in the process of adapting plants from sea to land. *MOCA1* homologous genes from lower to higher plants and from marine to terrestrial plants were selected for protein sequence comparison (**Figure 6B**). It was found that these homologous genes all contained a conserved domain and were α-helical regions. The amino acids in this domain change from hydrophilic amino acids such as serine and glycine to hydrophobic amino acids such as valine and alanine. The higher the plant, the more hydrophobic amino acids, and the lower the affinity for water molecules. This is crucial for the formation of alpha-helix regions and may make plants increasingly sensitive to Na^+^. From *Chlamydomonas reinhardtii* to higher plants, *Arabidopsis* and *Oryza sativa*, the hydrophobicity of amino acids in conserved domains increases, which is very important for plants to avoid adverse environments.

### 3.7 The response of *MOCA1* homologous transgenic plants to salt stress

**Figure 7:**
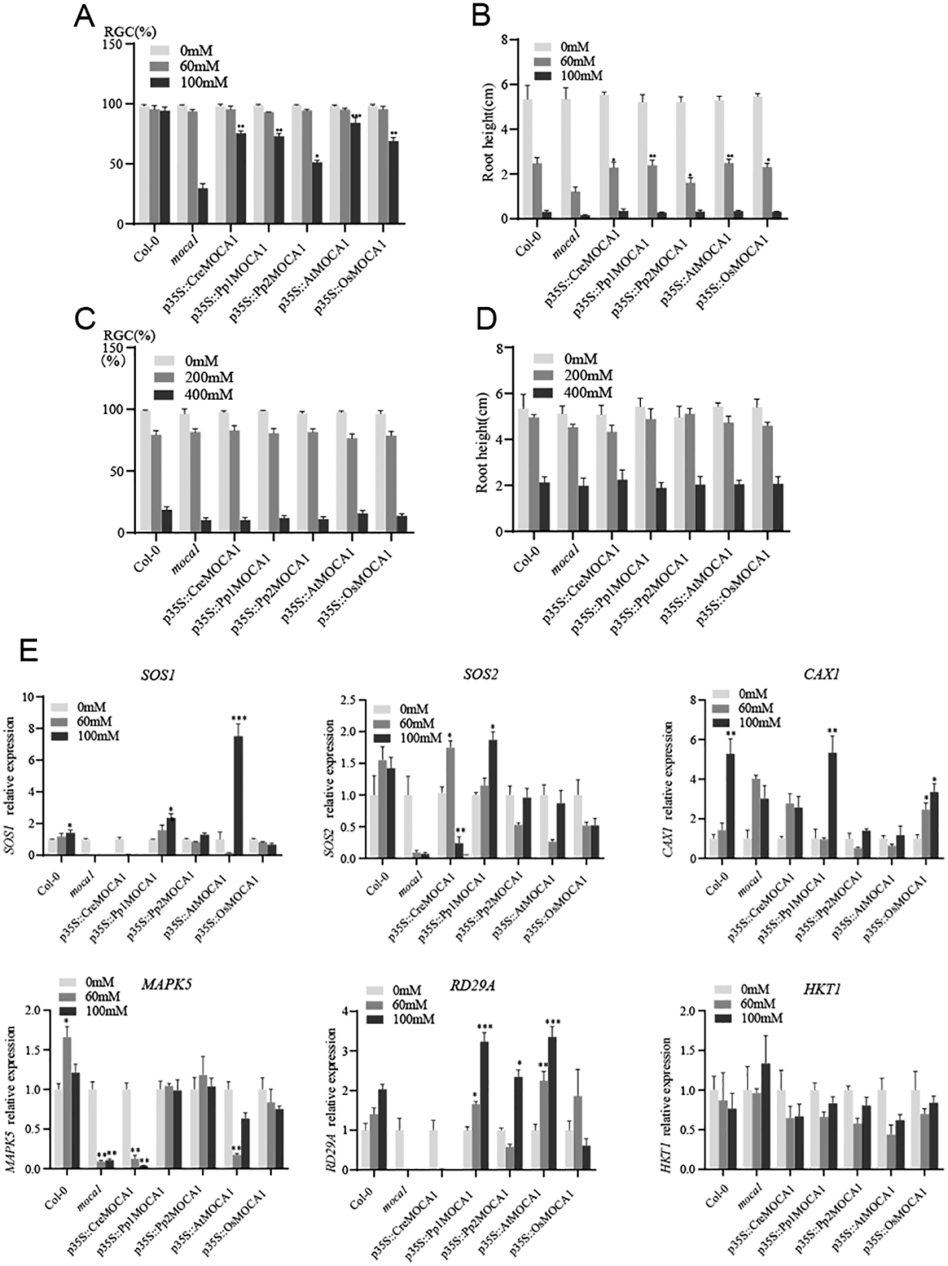
Response of *MOCA1* homologous genes to salt stress. The *MOCA1* homologous gene was introduced into the *moca1* mutant to generate complementary transgenic plants. **(A)** The ratio of green cotyledons after 10 days of growth in transgenic plants under 0, 60, and 100 mM NaCl treatments. **(B)** Root length of plants corresponding to **(A, C)** The ratio of green cotyledons after 10 days of growth in transgenic plants treated with 0, 200, and 400 mM sorbitol. **(D)** Root length of plants corresponding to **(C)**. The data represent the mean ± s.d. (*n* = 6). **(E)** Expression of salt-responsive gene in transgenic plants. qRT-PCR was used to determine the relative expression levels of six salt-responsive genes under salt stress. Seven-day-old *Arabidopsis* plants were treated with different concentrations of NaCl (0, 60, and 100 mM) for 6 hours to analyze the expression patterns of salt-responsive genes. The expression level of *Col-0* was set to 1 at 0 mM NaCl. Each group of experiments was repeated three times. The data represent the mean ± s.d. *P < 0.05, **P < 0.01, ***P < 0.001.

We constructed transgenic *Arabidopsis* plants by cloning the homologous genes *Cre_10g422450v5*, *Pp3c2_32110*, *Pp3c1_6500*, and *loc_Os02g41520.1* of the *MOCA1* genes in *Chlamydomonas reinhardtii*, *Physcomitrella patens*, and *Oryza sativa*. Comparing the root length and RGC index of the plants under different salt treatments, it was found that the five restorer lines had different degrees of resistance to salt stress, especially significant compared with the *moca1* mutant (**Figures 7A, B**). In order to know whether they have resistance to osmotic stress, they were treated with different concentrations of sorbitol (**Figures 7C, D**). The findings demonstrated no discernible differences in green cotyledons and root development compared with the wild type, revealing that the *MOCA1* homologous gene overexpression is insensitive to osmotic stress but has certain tolerance to salt stress. Remarkably, overexpression of the homologous gene *CreMOCA1* in *Chlamydomonas reinhardtii* can also restore root development, and the two homologous genes in *Physcomitrella patens* have differences in tolerance to salt stress.

Plants respond rapidly after being exposed to salt stress, including the efflux of Na^+^, the MAPK cascade reaction, and the storage by vacuoles (Waheed et al., 2024; Liu et al., 2025b). We analyzed salt-responsive gene expression to investigate whether the *MOCA1* homologous gene has the function of improving salt stress response (**Figure 7E**). The findings demonstrated that the expression of the Na^+^/H^+^ antiporter *SOS1* gene in the *moca1* mutant decreased under different concentrations of salt stress, and its expression also decreased in transgenic plants of the *Chlamydomonas reinhardtii MOCA1* homologous gene. However, its expression in the *MOCA1* transgenic plants of *Physcomitrella patens* increased significantly under salt stress, and its expression in the *Arabidopsis MOCA1* gene restorer line plants was significantly higher compared to others. *RD29A* is a gene that responds to salt stress, and its overexpression can significantly improve the salt resistance of plants. Under salt stress, the *RD29A* gene in *Pp1MOCA1* and *AtMOCA1* plants increased significantly compared with other plants. In short, salt-responsive gene expression in transgenic plants with *MOCA1* homologues was increased compared with the *moca1* mutant under salt stress, which is helpful to enhance salt tolerance.

### 3.8 Detection of Ca^2+^ in transgenic plants under salt stress

**Figure 8:**
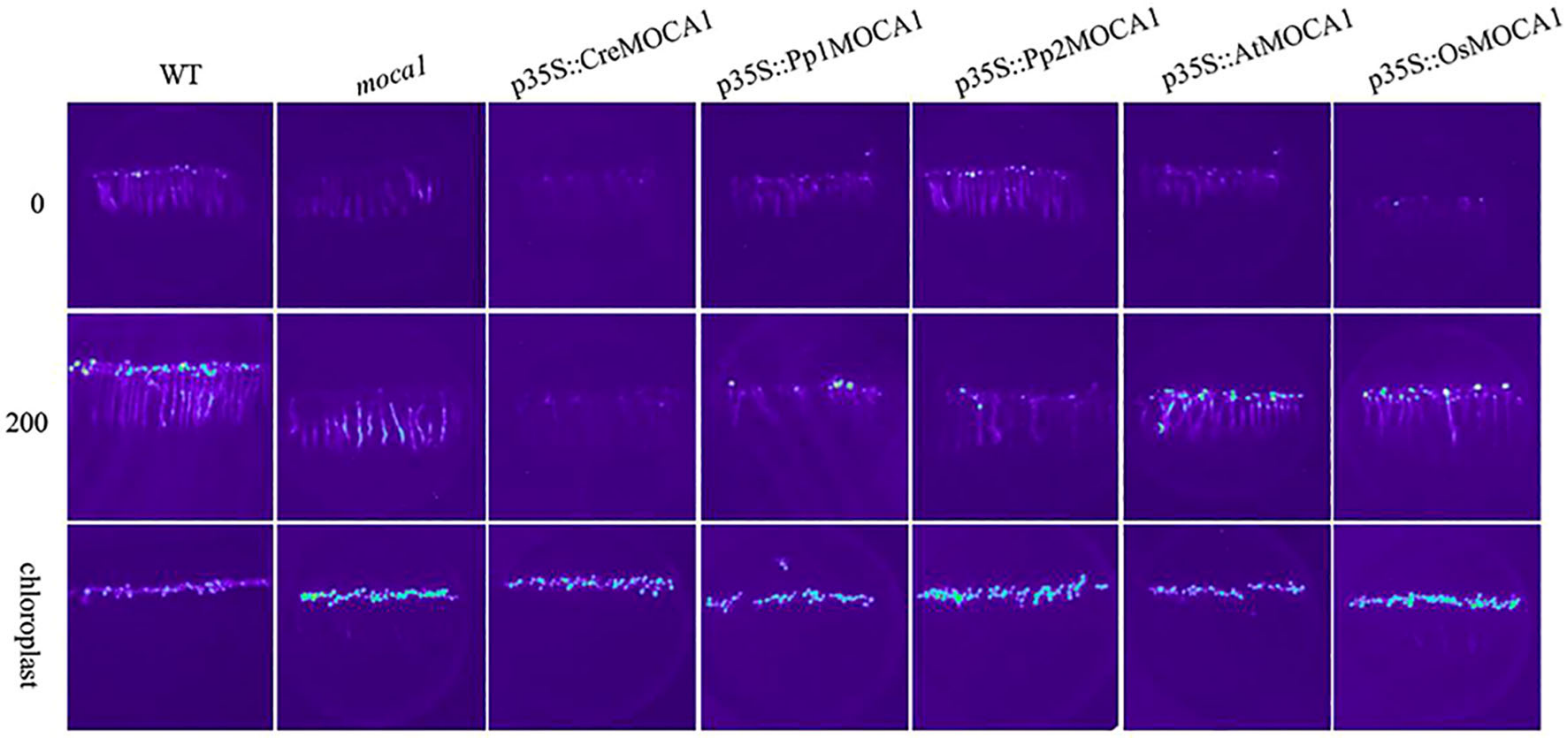
Detection of Ca^2+^ in transgenic *Arabidopsis thaliana* under salt stress. Ca^2+^ imaging technology based on aequorin bioluminescence was used to detect changes in intracellular calcium ion concentration. Different transgenic plants were treated with 0 or 200 mM NaCl, and changes in calcium ion signal were recorded. *Arabidopsis* chloroplast fluorescence was used to indicate that the plant had normal function. Each group of experiments was repeated at least three times.

Salt sensing in *Arabidopsis* is due to the combination of external Na^+^ with GIPC sphingolipid anions located on the plasma membrane, which opens the calcium ion channel, and a large amount of extracellular Ca^2+^ influx to trigger intracellular Na^+^ efflux or vacuole storage. Therefore, the detection of calcium ion signals is crucial for salt-sensing research. Ca^2+^ imaging based on aequorin bioluminescence can well detect the changes of intracellular calcium concentration. In transgenic plants aequorin can recombine and can exhibit Ca^2+^ increases resulting from salt, dryness, cold, and oxidative stress (Shen et al., 2025). We detected the calcium ion signals of different transgenic plants treated with 200 mM NaCl (**Figure 8**). Within 2 minutes after being stimulated by Na^+^, the wild-type plants’ Ca^2+^ changed dramatically. On the contrary, there was no significant change in Ca^2+^ after the *moca1* mutant plants were stimulated by exogenous Na^+^, which is consistent with the previous results (Jiang et al., 2019). This suggests that the *moca1* mutant exhibits sodium insensitivity, indicating that the sensing of Na^+^ in plants depends on the normal expression of the *MOCA1* gene. *CreMOCA1* restorer lines also had no signal detected, while *Pp1MOCA1* detected a calcium ion signal, but the signal was weak. *AtMOCA1* completely restored calcium ion signaling, which may be due to the fact that the plant’s *MOCA1* gene was highly expressed. Although *OsMOCA1* was also detected, the signal was weak compared to *AtMOCA1* plants. It is worth noting that among the two homologous genes of *MOCA1* in *Physcomitrella patens*, *Pp2MOCA1* has almost no calcium ion signal detected. We speculate that it may be distantly related to the *MOCA1* gene and does not yet possess a complete salt-sensing function.

### 3.9 Comparative analysis of ROS levels in transgenic plant root tips

**Figure 9:**
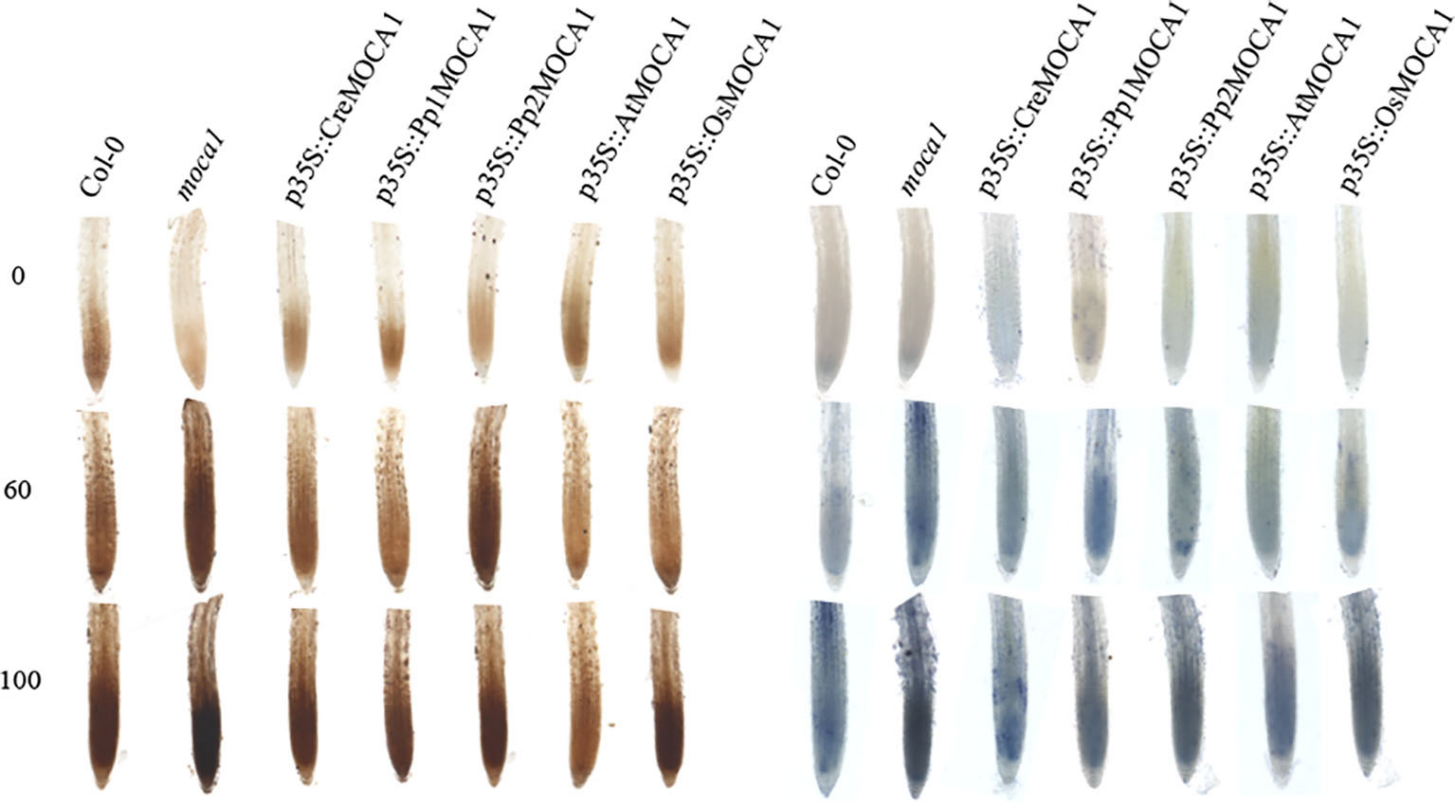
Assessment of ROS levels in the root tips of transgenic *Arabidopsis* plants under salt stress. Transgenic *Arabidopsis* plants were treated with different concentrations of NaCl (0, 60, and 100 mM) to assess ROS levels in their root tips. DAB staining on the left and NBT staining on the right. Each group of experiments was repeated three times, and no less than 30 roots were selected.

As a signal hub, ROS plays a very important role in signal transmission, so understanding how ROS changes in plant root tips is crucial (Kesawat et al., 2023; Jiang et al., 2025). Under varying salt stress concentrations, significant differences in ROS content were observed in the roots of wild-type plants, the *moca1* mutant, and multiple *MOCA1* homologous transgenic lines (**Figure 9**). The *moca1* mutant shows the accumulation of ROS in roots, which increases with increasing salt concentration. This may be related to its insensitivity to Na^+^, which affects the normal ROS signal transduction pathway, suppresses the expression of related genes, and fails to clear superoxide anions and hydrogen peroxide produced by stress in time. Further analysis found that the accumulation of ROS in the roots of all transgenic plants was considerably lower than in the *moca1* mutant. *AtMOCA1* has the strongest clearance ability due to the overexpression of *Arabidopsis thaliana*’s own genes. Although *CreMOCA1* has little signal in calcium ion detection, it still has the ability to clear excess ROS. *MOCA1* homologous transgenic *Arabidopsis* can clear excess ROS, thereby alleviating the damage to roots caused by oxidative stress due to high salinity.

### 3.10 Tissue expression

**Figure 10:**
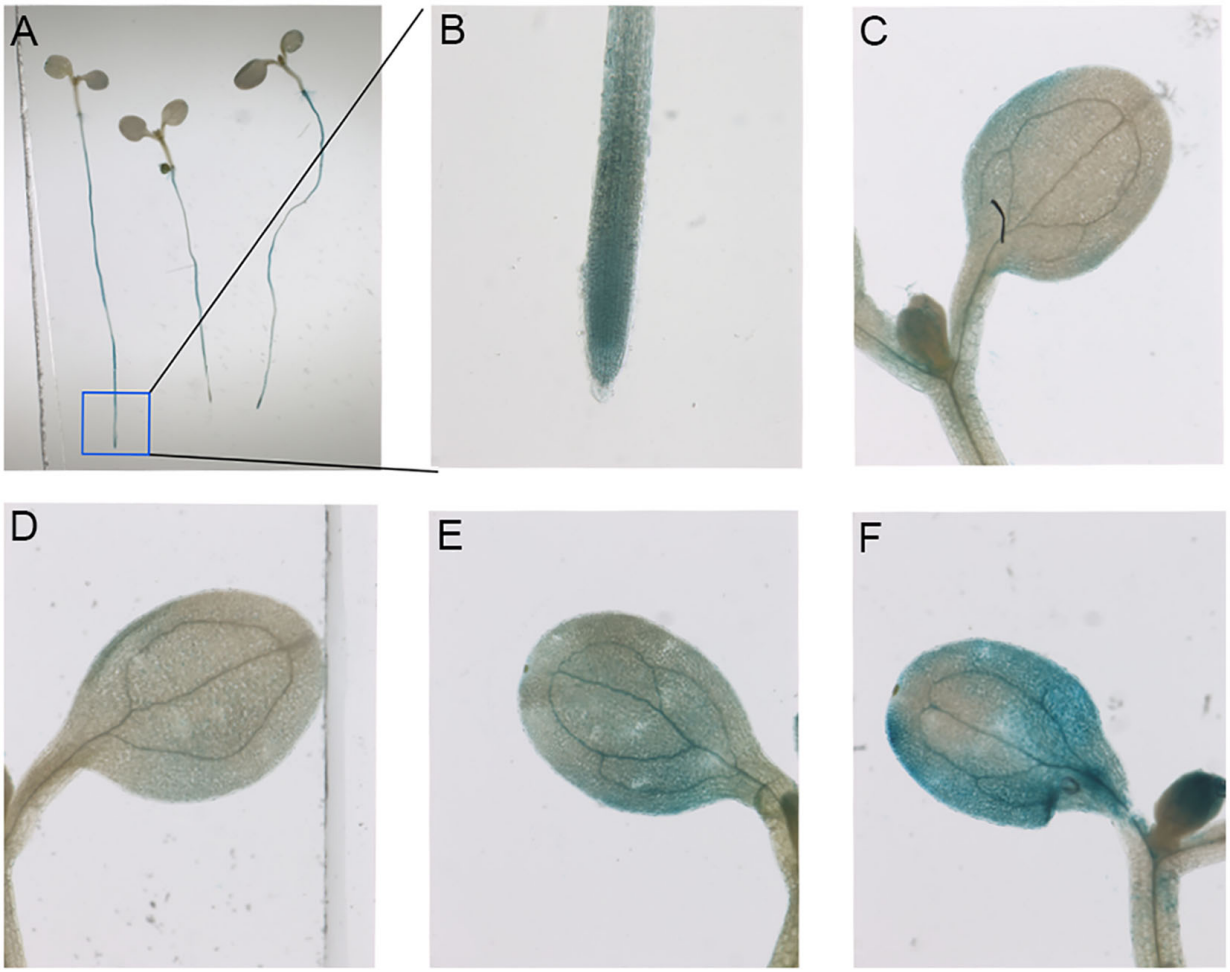
Tissue-specific expression of *proCreMOCA1::GUS* in transgenic *Arabidopsis* plants under salt stress. **(A)** Tissue staining of *GUS* genes in transgenic *Arabidopsis thaliana* plants grown under normal conditions for 7 days. **(B, C)** Tissue staining of *GUS* genes in roots and leaves of *Arabidopsis thaliana* under normal growth conditions. **(D-F)** Tissue staining of *GUS* genes in leaves treated with 0, 50, and 100 mM NaCl. Each group of experiments was repeated three times.

β-glucuronidase (GUS) could react with X-Gluc to produce a blue substance under certain circumstances. The substance is insoluble in the transgenic nucleus and visible under a microscope (He et al., 2020). To investigate whether the early *MOCA1* homologous gene’s promoter has certain salt-sensing ability and specific tissue expression localization, we established transgenic plants with fusion expression of *proCreMOCA1* and *GUS*. It was found that the *CreMOCA1* gene was mainly expressed in root tips and leaves of *Arabidopsis* (**Figure 10**). As the salt content rises, the blue color of the leaves becomes darker, indicating that the rise in salt concentration induces the *CreMOCA1* gene to elevate expression.

## 4 Discussion

Compared with *MOCA1*, *PGSIP7* responded to salt stress, whereas *PGSIP8* did not. The *pgsip7* mutant showed significantly inhibited root elongation and cotyledon development under salt stress compared to *pgsip8*. The SOS signaling pathway is essential for plant responses to excessive intracellular Na^+^ (Zhao et al., 2021; Liu et al., 2025a). No substantial change was found in the expression of the Na^+^/H^+^ antiporter *SOS1* between the *pgsip8* mutant and wild type under salt stress, indicating that it can normally efflux excess Na^+^. However, multiple salt-responsive genes in *pgsip7*, including *SOS2*, *MAPK5*, and *RD29A*, showed significant suppression. The *pgsip7* mutant exhibited progressive superoxide anion accumulation in roots with increasing salt concentration. It should be noted that the staining in *pgsip8* at 60 mM reflects normal, transient ROS signaling that is effectively cleared, fundamentally distinct from the failed ROS clearance and progressive accumulation in salt-hypersensitive mutants (*pgsip7* and *moca1*). Salt stress induced *MAPK5* upregulation in wild-type plants, but this response was abolished in *pgsip7* and *moca1* mutants, particularly at 100 mM NaCl. This inverse correlation between elevated ROS and reduced *MAPK5* expression suggests that impaired MAPK5 function may be associated with compromised ROS scavenging capacity. Previous studies have demonstrated that MAPK cascades are known regulators of ROS homeostasis. For instance, tomato SlMAPK3 functions as a positive regulator of salt tolerance by enhancing antioxidant enzyme activities (POD, SOD, CAT, APX), and its deficiency leads to H_2_O_2_ accumulation (Shu et al., 2022). In *Arabidopsis*, the MEKK1-MKK1/2-MPK4 pathway maintains ROS homeostasis through the regulation of antioxidant enzyme genes such as *CAT2* and *tAPX*, and disruption of this pathway results in ROS accumulation and cell death (Pitzschke et al., 2009). Similarly, rice OsMPK15, albeit functioning as a negative regulator of immune responses, also modulates ROS levels, as its loss enhances chitin-induced ROS burst (Hong et al., 2019). Although these kinases are not orthologous to MAPK5, they collectively illustrate a conserved framework of MAPK-mediated ROS regulation. We hypothesize that suppressed *MAPK5* in *pgsip7* and *moca1* disrupts antioxidant defense, perturbing ROS balance and causing oxidative stress. Although *pgsip8* has a similar protein structure to *moca1*, it is not sensitive to salt stress. This could be due to the insertion position of the mutant T-DNA on the intron, without silencing gene expression. The current study knows little about the functions of the *PGSIP7* and *PGSIP8* genes. With the discovery of *MOCA1* function, research on their functions may focus on the study of Na^+^ sensing (Jiang et al., 2019). Genome transfer in unicellular plants is one of the reasons for the evolution of gene functions. It may also be due to the duplication of family genes, resulting in functional redundancy among multiple genes. The *pgsip7* mutant exhibits sensitivity to Na^+^. However, it remains unclear whether this sodium intolerance is due to the absence of functional calcium ion signaling pathways. Therefore, this question will be a focus of future research.

Na^+^ are particularly vital for plant cells. The uneven distribution of salt in the soil severely restricts plant growth and development (Valenzuela et al., 2022). The discovery of the *MOCA1* gene explains how plants sense Na^+^. The members of the gene family to which *MOCA1* belongs are mainly involved in processes related to cell wall synthesis, such as xylan synthesis, glycosyl synthesis, and catalysis of galacturonosyl transfer. However, little is known about their specific functions (Kong et al., 2019). Most structures in plants that sense the external environment were discovered on the cell surface, including sensors such as osmotic, salt, and hydrogen peroxide (Yuan et al., 2014; Jiang et al., 2019; Wu et al., 2020). Cellulose synthase requires the participation of *AtPGSIP1* and *AtPGSIP3* genes in secondary cell wall synthesis (Yin et al., 2010). In the *pgsip7* mutant’s response to salt stress, it might be a catalytic enzyme in cell wall synthesis, and it is salt-sensitive by affecting cell wall synthesis. SOS2 activates the Na^+^/H^+^ antiporter SOS1 by binding to the SOS3 protein (Zhang et al., 2023a; Zhang et al., 2023b). The double mutant *pgsip7 sos2* seems more sensitive to salt stress than the others, similar to the *sos2* single mutant, which may indicate that the *PGSIP7* gene participates in the SOS signaling pathway.

Early unicellular organisms lived in the ocean. With the emergence of land, the adaptability of organisms from ocean to land has been continuously enhanced. During this process, the evolution of salt-sensing ability is one of the key factors for higher plants to survive (Wang et al., 2022; Zhou et al., 2024). Even though the early ocean’s salt content was slightly lower than that of today, it was still salt water. When plants appear in the terrestrial environment, they become diversified and coordinated in function to adjust to the complex and changeable circumstances. Large-scale evolution from simple green algae to complex multicellular terrestrial plants has resulted in the emergence of a wide variety of tissues and organs (Graham, 1996; McCourt et al., 2023; Madhani and Nejad Kourki, 2024). Higher plants have evolved various sensors that sense external stimuli. These sensors can keenly sense changes in the external environment so that plants can further respond and maintain normal growth and development (Albert et al., 2019).

Terrestrial plants have different ways of regulating Na^+^. Lower plants, especially mosses and ferns, control Na^+^ contents in the body by controlling inflow and outflow. However, Na^+^-ATPase does not seem to be expressed in higher plants (Chen et al., 2022), and they transport excess Na^+^ out of the cytoplasm or store it in vacuoles through the Na^+^/H^+^ antiporters (Yang et al., 2023; Zhang et al., 2023a). *MOCA1* catalyzes GlcA transfer to the phosphate side of IPC, forming the sphingolipid GIPC on the outside of the plasma membrane. Under salt stress, the negative charge carried by GIPC combines with Na^+^ to open calcium channels, and extracellular Ca^2+^ enters the cell to activate the downstream SOS signaling pathway (Jiang et al., 2019). The regulation of Na^+^ concentration in lower plant cells is different from that in higher plants, which may mean that their ability of salt-sensing is lower than that of higher plants. The conserved domains of *MOCA1* homologous proteins of *Chlamydomonas reinhardtii* and *Physcomitrella patens* are mostly hydrophilic amino acids, which have a great impact on the alpha helix of the protein, resulting in changes in the tertiary structure. Although the *MOCA1* gene of lower plants *Chlamydomonas reinhardtii* and *Physcomitrella patens* enhanced salt stress resistance, it did not restore the ability to sense Na^+^. Plants’ Na^+^ sensing ability may be gradually acquired during evolution due to the adaptation mutation of amino acid sites in conserved domains. This complex process needs further research.

While our phylogenomic and transgenic analyses outline a stepwise acquisition of *MOCA1*-dependent Na^+^ sensing, the present study has two main limitations. First, the conclusion that ROS accumulation underlies the salt-hypersensitive phenotypes of *moca1* and *pgsip7* rests solely on histochemical staining; quantitative transcript levels of ROS-producing and ROS-scavenging genes (e.g., *RbohD*, *RbohF*, *APX1*, *CAT2*, *FSD1*) in the mutant lines remain to be determined, and the direct causal relationship between *MAPK5* down-regulation and ROS accumulation has not been established through genetic and biochemical means, nor has the distinction between protein expression levels and phosphorylation activity been addressed. Second, the causal link between these ROS changes and the observed growth inhibition has not been functionally validated. Future work should therefore start with qPCR or RNA-seq profiling of the above ROS-hub genes in *moca1*, *pgsip7* and *pgsip8* under NaCl stress to test whether transcriptional reprogramming correlates with ROS accumulation, and simultaneously examine MAPK5 kinase activity and the expression regulation of its downstream antioxidant defense genes. Subsequently, generation of *mapk5* knockout lines to phenocopy the oxidative stress profile and stable CRISPR/Cas9 knockout or over-expression of the ROS-hub genes in the *moca1* and *pgsip7* backgrounds, particularly the construction of *MAPK5* over-expression lines to verify whether they can rescue the ROS phenotypes of the mutants, coupled with quantitative ROS imaging and root-growth assays, will directly assess their genetic contribution to salt tolerance. Subcellular-localization experiments using fluorescent protein fusions should further clarify where the respective ROS-producing and ROS-scavenging proteins operate (plasma membrane, peroxisome, cytosol), and yeast two-hybrid or co-immunoprecipitation assays should be employed to detect the interaction between MAPK5 and MOCA1/PGSIP7, followed by epistasis analysis using double mutants. These complementary expression and functional studies will provide the mechanistic evidence required to confirm that the proposed evolutionary gain of *MOCA1*-dependent Na^+^ sensing is coupled to the transcriptional re-wiring of ROS-producing and ROS-scavenging modules and elucidate the central role of MAPK5 in this regulatory network, thereby deepening our understanding of how plants fine-tune oxidative signaling to cope with saline environments.

## 5 Conclusions

This study focused on the response of *Arabidopsis* salt-sensing genes *MOCA1*, *PGSIP7*, and *PGSIP8* to salt stress and the evolution of the *MOCA1* gene to explore potential salt-sensitive gene functions and the acquisition of the *MOCA1* salt-sensing function. Through bioinformatics analysis of *GT8* subfamily members, two potential salt-sensitive genes, *PGSIP7* and *PGSIP8*, were identified. Through the analysis of the mutant’s phenotype, ROS content, and the expression of the salt-responsive gene, it was revealed that the *PGSIP7* gene is responsive to salt stress and may participate in the SOS signaling pathway. In addition, this study started from the evolution of the *MOCA1* gene in lower-to-higher plants, and through the comparison of homologous sequences among different species, it was found that the hydrophobic amino acids contained in the protein sequences of the *MOCA1* homologous gene in lower-to-higher plants gradually increased, consistent with their phylogenetic relationships. Transgenic plants with the *MOCA1* homologous gene in lower to higher plants were further constructed by cloning and expressing heterologous genes. The calcium ion signals in different transgenic *Arabidopsis thaliana* were detected by plant *in vivo* imaging technology. These findings illuminate the stepwise evolutionary trajectory by which plants acquired critical salt-sensing capabilities during the transition from aquatic to terrestrial environments, with the Na^+^ sensing ability of *MOCA1* homologs progressively enhanced from *Chlamydomonas reinhardtii* to *Physcomitrella patens* and further to *Oryza sativa* and *Arabidopsis thaliana*. Moreover, overexpression of these *MOCA1* homologs confers salt tolerance, suggesting promising strategies for engineering salt-tolerant crops to address soil salinization threatening global food security.

## Data Availability

The original contributions presented in the study are included in the article/**Supplementary Material**. Further inquiries can be directed to the corresponding author.
