# Supplementary material for: Evolutionary analysis of MOCA1 gene in Arabidopsis thaliana and functional comparison with PGSIP7 and PGSIP8 genes
Source: Front Plant Sci. 2026 Feb 24;17:1734646. doi: 10.3389/fpls.2026.1734646 (PMC12971971; doi:10.3389/fpls.2026.1734646)
Supplement: Supplementary file 1 [file Table1.docx]

Supplementary Material

# Supplementary Figures
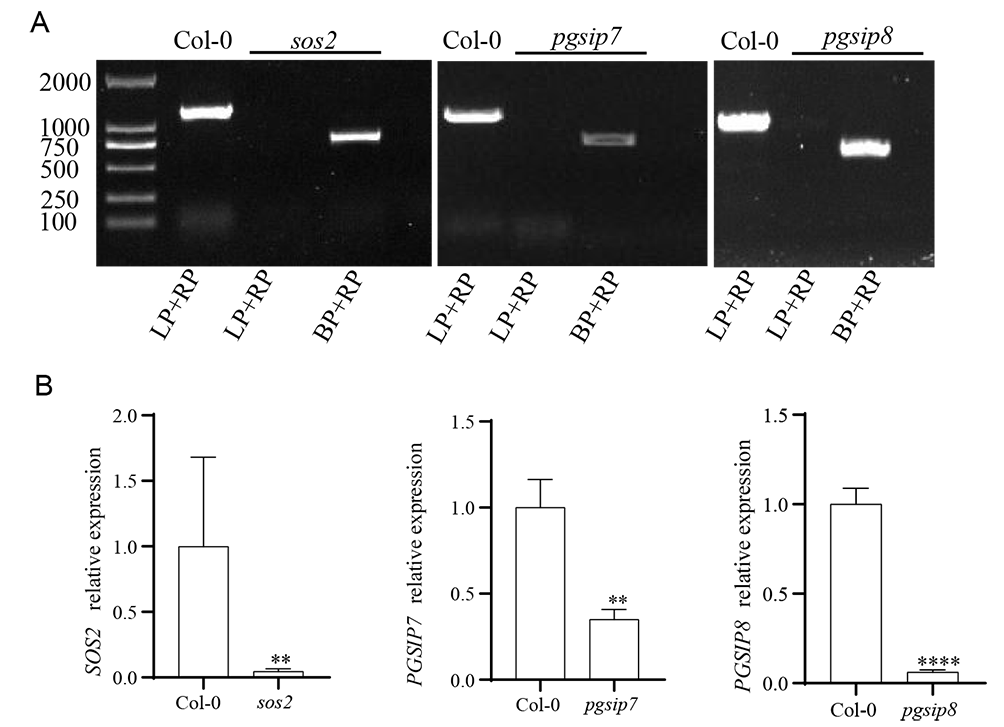


**Supplementary Figure 1.** Identification of *pgsip7*, *pgsip8*, and *sos2* mutants. (A) Identification of three mutants using the tri-primer method. (B) Relative expression level analysis. The bar chart displays the relative expression levels of the target gene in different mutant plants. The *Col-0* expression level was set to 1. The data represents the mean ± s.d.
